# How marital events explain the suicide rate among Chinese

**DOI:** 10.1371/journal.pone.0286961

**Published:** 2023-10-23

**Authors:** Xueyan Yang, Liping Liu, Rui Li

**Affiliations:** 1 Population and Development Studies, School of Public Policy and Administration, Xi’an Jiaotong University, Xi’an, Shaanxi, China; 2 Population and Development Studies, School of Public Policy and Administration, associate Professor at Sports Center, Xi’an Jiaotong University, Xi’an, Shaanxi, China; 3 Population and Development Studies, School of Public Policy and Administration, Xi’an Jiaotong University, Xi’an, China; Xiangtan University, CHINA

## Abstract

**Aims:**

The aims for this study was to prove the impacts of marital events (marriage rate, divorce rate and marriage squeeze), economic development, and social development on the suicide rate among urban and rural Chinese and reveal the differences in these impacts between urban and rural areas and between genders.

**Methods:**

An explanatory time-series analysis methodology was adopted to analyze the nation-wide data ranging from 1987–2017.

**Results:**

Marriage rate was a protective factor against the suicide rate among urban and rural men, and rural women; however, divorce rate was a protective factor against the suicide rate only among rural women. For the four groups, the economic development level measured by per capita GDP is a protective factor, while social development measured by urbanization and rural–urban labor migration rates in rural areas plays different roles.

**Conclusions:**

Marriage and divorce rates were found to have different meanings for the four groups. This study offers a reference for designing relevant policies and projects to intervene in suicidal behaviors among different groups.

## Introduction

### The changes in suicidal rate in China

**Fig 1:**
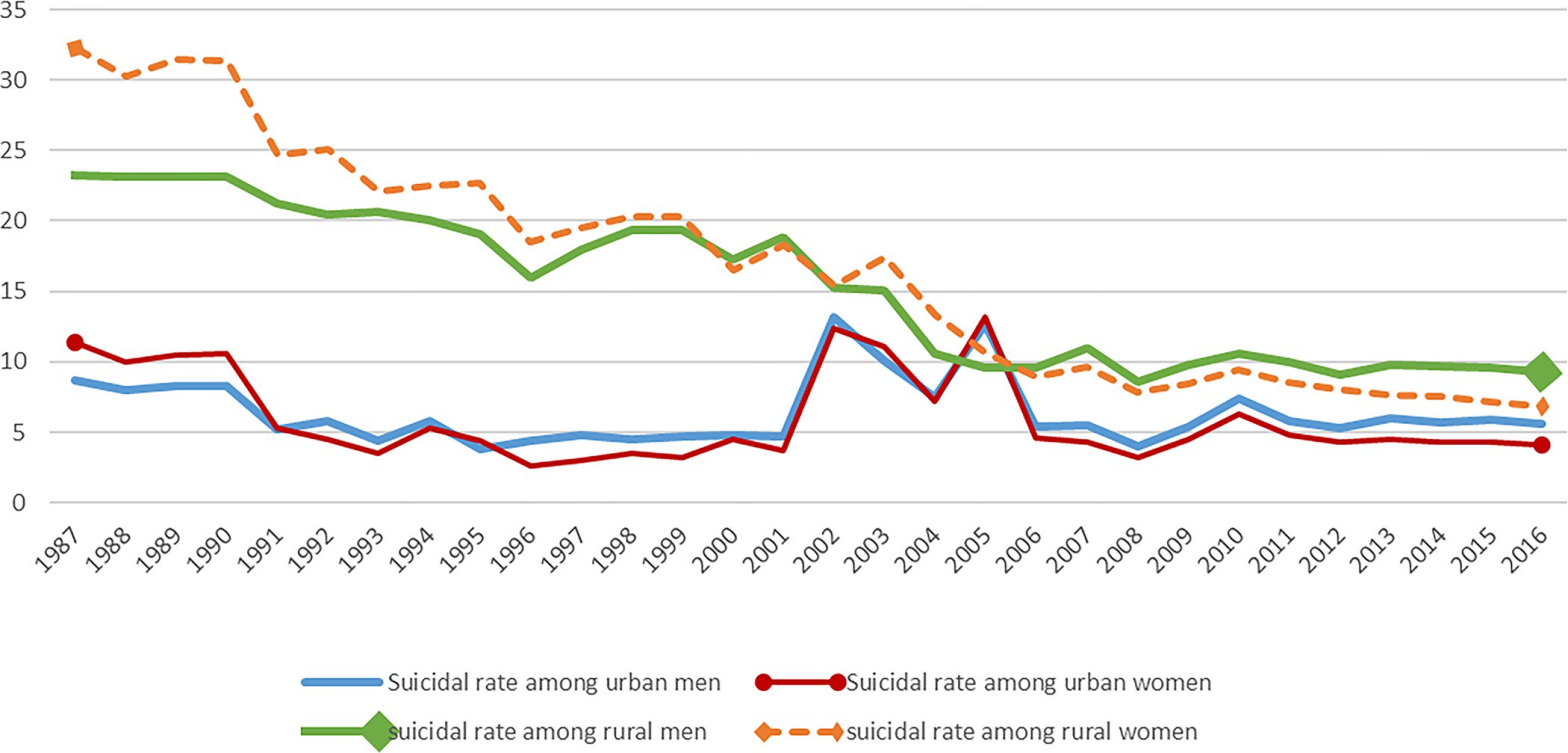
The suicidal rate by urban and rural areas and by genders 1987–2016. Note: The data 1987–2008 is from Zhang’s study (2011), and the data 2009–2016 is from China National Health Statistical yearbook.

Suicide was a self-termination of life and includes stages such as suicidal intention, attempted suicide, and suicidal death [1]. Suicide prevention was a social problem that requires urgent solutions and was a priority field in sociology and public health. According to a report by the WHO (2012), one person committed suicide every 40 s worldwide, and at least 20 million people had suicidal intentions. In the past 40 years, the suicide rate had increased by 60% globally, with most suicide cases reported in China due to its large population. As shown in Fig 1, contrary to the global trend in the suicide rate, the suicide rate of China had four important changes over the past 30 years. 1) An apparent 6/100 thousand decrease in the overall suicide rate, which was significantly lower than the global average level; 2) an apparent age-based pattern showing a higher suicide rate among the elderly than among the younger group; and 3) the gender pattern had changed. The suicide rate had changed from being higher to being lower among women than among men due to a decrease in the suicide rate among women. 4) Further, an apparent difference in suicide rates between urban and rural areas was noted. While the suicide rate had decreased more in rural areas than in urban areas, it was still higher in rural areas than that in urban areas [2].

China’s demographic and social development had a long-standing urban-rural dualism; for example, the fertility rate and sex imbalance level was lower in urban areas than it was in rural areas; however, the degree of aging was similar to, or lower than that in rural areas [3]. Meanwhile, the level of social security and public health services in urban areas was better and more complete than that in rural areas [4], which might cause the development of dualism in the suicide rate in urban and rural China. The process of reform and opening-up since 1980 had caused a huge change in China’s society; accordingly, the structure of social relations in traditional rural areas had greatly changed, which might had influenced people’s suicidal behaviors [5].

### The changes in marital events in China

Conversely, the marital events represented by marriage rate, divorce rate and marriage squeeze had changed in transitional China. The marriage rate had developed in a zigzag manner, from 8.7‰ in 1987, which first decreased to 6.3‰ in 2005, increased to a peak value of 9.6‰ in 2013, and then decreased to 7.7‰ in 2017; simultaneously, the divorce rate increased from 0.7‰ in 1987 to 3‰ in 2017 [6]. Meanwhile, as most parents prefer sons and widely use B-ultrasonic technology, China had experienced a more highly distorted sex ratio at birth (SRB) with the longest time, deepest extent, and widest scope, reaching a peak value of 120.56 in 2005 and then slowly decreasing. Since 2017, the SRB had still fluctuated around 111.9 [7]. A higher SRB led to an imbalanced sex structure in adulthood and a severe marriage squeeze in the marital market [8]. It was estimated that about 10% of the surplus men hardly find enough women to marry, and the total number of surplus men in China was about 33 million [9]. Owing to the universal marriage pattern (namely that societies in which majority of people believe that “everybody should get married” and they practice this concept) and hypogamy marriage model, marriage-squeezed men mostly lived in remote rural areas and poverty, lacked social resources, and easily experienced the dual pressures from family and society [10].

The existing studies stated that, as the most intimate personal relationship, marital events including marriage, divorce and marriage squeeze definitely had an impact on suicidal behavior, which varied across genders [11]. For example, the existing literature found that, for men and women, marriage was a protective factor and a risk factor for suicide, respectively [12–14]. Therefore, how did changes in marital events influence the suicide rate among rural and urban Chinese in the dual system? Did the unstable development in marital rate, the stable increase in the divorce rate, and the marriage squeeze increase or reduce suicidal behaviors among Chinese people? These questions had not been systematically answered before.

## Research context

### Evidence before this study

#### Geographical evolution of studies on suicide

Studies on suicide had long developed, from psychology to sociology, among which the most classic study was “the suicide theory” by Durkheim. In this book, Durkheim adopted the empirical sociology methods to comprehensively analyze human beings’ suicidal behaviors and found that suicide was associated with social facts, including nationality, natural environment, gender, marital status, religion, stability, and prosperity of society, while the fundamental factors for suicide rate were social integration and moral rules [11]. Later, researchers attempted to explain human suicidal behaviors based on psychology and sociology paths [1]. In early years, sociologists recognized that, as the most crucial content in personal relationships, marital relationships definitely had an impact on suicidal behaviors. Durkheim also proved the relationship between marriage and suicidal behaviors. He pointed out that marriage can reduce suicidal risks, especially for women; single men are more likely to experience suicidal risks, and divorce could increase suicidal risks and behaviors among men but reduce them among women [11]. This suggested that marriage and divorce might have different functions and impacts on suicidal behaviors for men and women. Studies on French people for the period 1981–1993, as well as a study from Hongkong women supported Durkheim’s viewpoints [15,16]; However, most studies proved that the marriage was a protective factor while divorce was a risky factor for suicidal behavior no matter what the gender was [17–21] and this suggested that the relationship between marriage and suicidal behaviors might be expressed differently in different societies and cultures. Based on the above existing research, it could be inferred that the relationship between marital events and suicidal behaviors was determined by the quality and people’s perception of marriage. If marriage was viewed as a strong social tie and social support, negative marital events such as divorce and widowhood would increase the suicide rate, and marriage would become a protective factor for suicidal behaviors [22,23]. If marriage was viewed as a constraint, pressure, or shackle, divorce would help release pressure and become a protective factor for suicidal behaviors [5,12–14].

#### Chronological evolution of studies on suicides in China

Since 1990’s, scholars had recognized the unique gender patter and change trends in suicidal rate in China and attempted to explain the suicidal behaviors of Chinese people with localized concepts and theories, including living theory by Wu Fei [24–27], migration theory by Jing Jun [5], strain theory by Zhang Jie [28], and motivation theory by Liu Yanwu [27,29]. Among those research, the marital factors were indicated to be connected with migration and suicidal behaviors [5]. The migration actually provided chances for rural women, who could migrate to urban areas and realized their own economic and marital independence. Once they were dissatisfied with their marriage, they may chose divorce to regain freedom rather than commit suicide, that’s why the suicidal rate were apparently declined among rural women in recent years [28,30]. This finding also supported Durkheim’s viewpoints from another perspective.

#### Other marital system that affects Chinese

To date, the marital system that affected Chinese’s daily lives and health had two types of culture. The first was “universal marriage system,” meaning that most Chinese people believe that “everyone should get married,” and people who cannot get married might experience the dual pressures from family and society, which may then affect their health [10,31]. The second was the “hypergamy system,” meaning that women tend to marry men who had higher socioeconomic statuses, and men with lower socioeconomic statuses were easily marriage-squeezed in the marital market [8,32]. Owing to hypergamy, marriage-squeeze problems existed throughout China’s history; however, the extent of the marriage squeeze was amplified by the surplus men because of the sex imbalance in the marital market since 1980 [33].

Although many studies had proved the relationship between marriage, divorce and suicide, which is uncertain, and no relevant study investigated the relationship between marriage, divorce, marriage squeeze and suicidal behaviors in Chinese, the existing studies suggested that social transition influenced the decrease in suicide rate; however, they only focused on the impacts of economic growth and migration while neglecting the possible impacts of marriage rate, divorce rate and the marriage squeeze due to sex imbalance on the suicide rate [28,30]. This study analyzed the impact of marital rate, divorce rate, and marriage squeeze on the suicide rate among Chinese people to explain Chinese suicidal behaviors.

### Added value of this study

To our knowledge, this was the study that tried to explain the suicide rate among Chinese, using marital events represented by marriage rate, divorce rate and marriage squeeze and also utilized the existing research most of which only focuses on the economic and social development.

### Implications of all the available evidence

An explanatory time-series analysis methodology was adopted in this study to prove the impacts of marriage rate, divorce rate, marriage squeeze on the suicide rate among urban and rural Chinese and reveal the differences in these impacts between urban and rural areas and between genders. This study’s practical implication was to reveal the direct impacts of different marital events on suicidal behaviors, which will offer a reference for designing relevant policies and projects to intervene suicidal behaviors among different groups.

## Data and methods

To examine the impact of marital events on Chinese suicidal behaviors, a gender-wise data on suicide rates in urban and rural areas was adopted in this study. First, a framework was proposed for time-series explanatory analysis. Further, the impacts of marital rate, divorce rate, sex imbalance, per capita GDP (Gross Gomestic Product), urbanization rate, and rate of rural–urban labor migration in rural areas on the suicide rate were analyzed by urban and rural areas, and gender. The scale of economic development was commonly measured by GDP, while the per capita GDP was more widely used and regarded as a measurement for the relative scale of economic development [28]. Therefore, here we used the per capita GDP to measure the economic development. Social development is a very complicated concept, which was covering many social aspects such as population, environment, resident’s living, employment, social security, health, education and technology [4]. In China, the urbanization had changed social development very deeply and comprehensively and was regarded as a crucial event to suicidal rate [4,5]. Therefore, to make it simple, the urbanization rate was adopted to measure the social development in urban areas, and the rate of rural-urban labor migration in rural areas was adopted to measure the social development in rural areas (as shown in Table 1).

**Table 1:** The definitions of key variables.

Variables	Definitions
Suicidal rate in urban men (UMSR)	The number of death men due to suicide to the number of total of urban men within a certain period (commonly means one year), and is commonly expressed by per 100000.
Suicidal rate in urban women (UWSR)	The number of death women due to suicide to the number of total urban women within a certain period (commonly means one year), and is commonly expressed by per 100000.
Suicidal rate in rural men (RMSR)	The number of death men due to suicide to the number of total rural men within a certain period (commonly means one year), and is commonly expressed by per 100000.
Suicidal rate in rural women (RWSR)	The number of death women due to suicide to the number of total rural women within a certain period (commonly means one year), and is commonly expressed by per 100000.
Marriage rate (MR)	The number of population who are getting married to the number of total population with a certain period (commonly means one year), and is commonly expressed by per 1000.
Divorce rate (DR)	The number of population who are divorced to the number of total population with a certain period (commonly means one year), and is commonly expressed by per 1000.
Sex ratio (SR)	The number of men population to number of women population and multiply by 100 in a certain area within a certain period (commonly means one year), and which is commonly fluctuating around 100.
per capita GDP (PGDP)	Per-capita gross domestic product within a certain period (commonly means one year) by the permanent residents in thin country
Urbanization rate (UR)	The number of population who live in urban areas to the number of total population within a certain period (commonly means one year)
Rate of rural-urban migration labors in rural areas (RUMR)	The number of rural-urban migration labors to the number of total labor population in rural areas within a certain period (commonly means one year), which is commonly expressed by percentage. The rural-urban migration labors means the rural labors who migrate to urban areas rather than their township for work; and the labors means the population aged 16–60 and having labor-force.

The data used in this study were from second-hand literature, the statistics data were from China’s government and relevant departments, and the details are as follows (as shown in Table 2):

1. The data on suicide rate from 1987 to 2008 were from the studies by Zhang Jie (2011) [28]; the data on suicide rate from 2009 to 2017 by urban and rural areas and by gender were from the China National Health Statistical yearbook.
2. The data on marital and divorce rates from 1987 to 2017 were from “An overview of the development of people’s livelihood” issued by China’s National Bureau of Statistics.
3. The data on sex ratio from 1987 to 2017 were from the population statistics bulletin by National Bureau of Statistics.
4. The data on the rate of rural–urban labor migration from 1987 to 2008 were from the study by Zhang Jie et al. (2011) [28], and the data on the number of labor migration in rural areas from 2009 to 2017 were from the population statistics bulletin by the National Bureau of Statistics. The data on the number of laborers in rural areas from 1987 to 2017 were estimated using the ratio of labor to total population from “China’s statistical yearbook on population and employment” and the total population in rural areas.
5. The data on China’s urbanization rate were obtained from the statistical bulletin by the National Bureau of Statistics. The migration rate in rural areas was adopted from Zhang Jie’s study to measure the proportion of rural-urban migration in the total population in rural areas [28]. However, this study included rural and urban populations, and the rural-urban migration may affect the population in urban and rural areas differently; therefore, the urbanization rate was used to measure the impacts of migration on the urban population and the rate of rural-urban migration in rural areas the measure the impacts of migration on the rural population.
6. The data on per capita GDP were obtained from China’s statistical yearbook.

**Table 2:** Statistical description of sample data.

	Observations	Mean	Medium	Min	Max	Std. Dev.
Suicidal rate in urban men (UMSR)	31	6.22	8.48	3.79	13.16	2.283
Suicidal rate in urban women (UWSR)	31	5.89	7.89	2.64	13.13	3.139
Suicidal rate in rural men (RMSR)	31	15.13	15.91	8.62	23.20	5.38
Suicidal rate in rural women (RWSR)	31	16.43	19.33	6.36	32.30	8.36
Marital rate (MR)	31	7.96	8	6.10	9.90	1.12
Divorce rate (DR)	31	1.42	1.87	0.53	3.20	0.79
Sex ratio (SR)	31	106.03	106	104.50	107.50	0.75
per capita GDP (PGDP)	31	18077.09	30161.85	1123.08	59200.61	17935.65
Urbanization rate (UR)	31	38.98	35.04	10.50	59.58	12.21
Rate of rural-urban migration labors in rural areas (RUMR)	31	18.48	21.73	1.95	41.51	13.40

**Fig 2:**
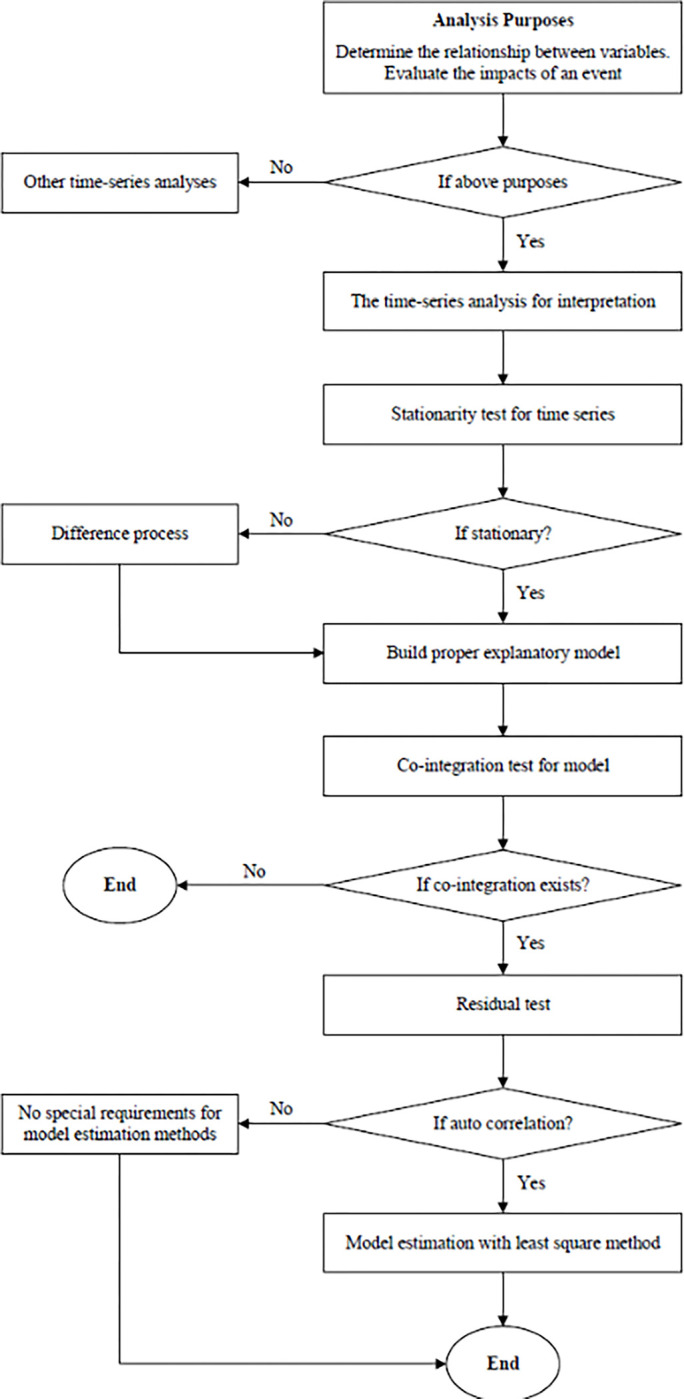
The steps for explanatory time-series analysis.

The analysis methods used for time-series data were relatively mature; in most social science fields, time-series data analysis was mainly used for prediction and, subsequently, complete steps and technologies were developed. In sociology and social psychology fields, time-series data analysis was used to explain a certain social phenomenon, rather than prediction, and the steps and methodologies adopted for this purpose differed from those for prediction. The analysis steps were in this present study based on the guidelines for time-series data analysis and suggestions from scholars, who proposed a specific step and methodology for time-series data analysis to explain study purposes [34]. Please find the flowchart in Fig 2.

## Results

### Stationary test for time-series data

To maintain stationary time series data, the natural logarithm was taken using time-series variables [35]. As shown in Table 3, these variables included men’s suicide rate in urban areas (UMSR), women’s suicide rate in urban areas (UWSR), men’s suicide rate in rural areas (RMSR), women’s suicide rate in rural areas (RWSR), marital rate (MR), divorce rate (DR), sex ratio (SR), urbanization rate (UR), per capita GDP (PGDP), and rate of rural–urban labor migration in rural areas (RUMR). Thereafter, the stationary test was conducted on all data using the ADF unit root method according to the scatter diagram Men’s suicide rate in urban areas (UMSR), women’s suicide rate in urban areas (UWSR), men’s suicide rate in rural areas (RMSR), women’s suicide rate in rural areas (RWSR), marital rate (MR), and per capita GDP (PGDP) passed the stationary test. Further, divorce rate (DR), rate of rural–urban labor migration in rural areas (RUMR), and sex ratio (SR) passed the stationary test after the first difference processing.

**Table 3:** The stationary test results on time-series data.

Variables	ADF	Test type (c,t,l)	1% threshold	5% threshold	10% threshold	Stationary
LNUMSR^(^^1^^)^	-3.495	(1,0,0)	-3.670	-2.964	-2.621	Yes
LNUWSR^(^^2^^)^	-2.852	(1,0,0)	-3.670	-2.964	-2.621	Yes
LNRMSR^(^^3^^)^	-2.682	(0,0,0)	-2.644	-1.953	-1.610	Yes
LNRWSR^(^^4^^)^	-1.704	(0,0,0)	-2.644	-1.953	-1.610	Yes
LNMR^(^^5^^)^	-4.053	(1,0,0)	-3.724	-2.986	-2.633	Yes
LNDR^(^^6^^)^	0.784	(0,0,0)	-2.647	-1.953	-1.610	No
D(LNDR)	-2.446	(1,0,1)	-2.647	-1.953	-1.610	Yes
LNSR^(^^7^^)^	-1.736	(1,1,0)	-4.297	-3.568	-3.218	No
D(LNSR)	-7.115	(1,1,1)	-4.310	-3.574	-3.222	Yes
PGDP^(^^8^^)^	-3.835	(1,1,0)	-4.339	-3.588	-3.229	Yes
UR^(^^9^^)^	-4.387	(1,0,0)	-3.670	-2.964	-2.621	Yes
RUMR^(^^10^^)^	-2.816	(1,0,0)	-3.670	-2.964	-2.621	Yes
LNRUMR	1.344	(1,1,0)	-4.356	-3.595	-3.234	No
D(LNRUMR)	-7.160	(1,1,1)	-4.356	-3.595	-3.234	Yes

Note

(1) Suicidal rate in urban men.

(2) Suicidal rate in urban women.

(3) Suicidal rate in rural men.

(4) Suicidal rate in rural women.

(5) Marital rate.

(6) Divorce rate.

(7) Sex ratio.

(8) per capita GDP.

(9) Urbanization rate.

(10) Rate of rural–urban labor migration to the total labor population in rural areas.

### Build explanatory models

According to the migration theory by Jing Jun and his colleagues, the existing studies on the relationship between marriage, divorce and suicide rate, and the assumptions on the relationship between marriage squeeze and suicide rate [5], a series of models were built by using an independent variable (suicide rate by area and gender), marital events variables (marital rate [MR], divorce rate [DR]), marriage squeeze variables (sex ratio [SR]), socioeconomic variables (urbanization rate [UR], per capita GDP [PGDP], and rate of rural–urban labor migration in rural areas [RUMR]). Owing to the urban and rural dualistic system, migration had different impacts on urban and rural areas. For urban areas, migration might induce an increase in the urbanization rate, and for rural areas, an increase in the rate of rural–urban labor migration [35]. Therefore, the last variable differed when the models were built for urban and rural areas, that was, the urbanization rate (UR) was included in the models for urban areas and the rate of rural–urban labor migration in rural areas (RUMR) was included in those for rural areas. The model equations were as follows:

$$lnUMSR=c_{01}+a_{1}lnMR+a_{2}D(lnDR)+a_{3}D(lnSR)+a_{4}lnPGDP+a_{5}(lnUR)$$ (1)

$$lnUWSR=c_{02}+b_{2}lnMR+b_{2}lnD(lnDR)+b_{3}D(lnSR)+b_{4}lnPGDP+b_{5}(lnUR)$$ (2)

$$lnRMSR=c_{03}+c_{1}lnMR+c_{2}D(lnDR‐1)+c_{3}D(lnSR)+c_{4}lnPGDP+c_{6}D(lnRUMR)$$ (3)

$$LnRWSR=c_{03}+d_{1}lnMR+d_{2}D(lnDR‐1)+d_{3}D(lnSR)+d_{4}lnPGDP+d_{6}D(lnRUMR)$$ (4)

In the above equations, D((lnDR), D(lnSR), and D(lnRUMR) were the first difference items after the natural logarithm was taken using divorce rate (DR), sex ratio (SR), and rate of rural–urban labor migration to the total labor population in rural areas (RUMR), respectively. The coefficients for each variable were a_1_-a_4,_ b_1_-b_4_ and c_1_-c_4_, d_1_-d_4_, and c_01,_ c_02,_ c_03,_ and c_04_ are constant terms.

### The Co-integration test for models

As shown in Table 4, Johanson’s co-integration test was adopted for the models, and the test results indicated at least two co-integration relationships in the models with UMSR and UWSR as the respective dependent variables. Further, at least five co-integration relationships were found in the models with RMSR and RWSR as the dependent variables, which indicated long-term equilibrium relations between the suicide rate by rural and urban areas and by gender and the five dependent variables.

**Table 4:** The Co-integration test results.

	Hypothesized No. Of CE(s)	Eigenvalue	Trace Statistic	0.05 Critical Value	Prob.**
Model 1	None *	0.881	144.586	95.754	0.0000
	At most 1 *	0.720	84.915	69.819	0.0020
	At most 2 *	0.604	49.241	47.856	0.0368
Model 2	None *	0.864	142.989	95.754	0.0000
	At most 1 *	0.752	87.100	69.819	0.0011
	At most 2 *	0.583	48.096	47.856	0.0475
Model 3	None *	0.930	171.459	95.754	0.0000
	At most 1 *	0.771	97.217	69.819	0.0001
	At most 2 *	0.542	55.999	47.856	0.0071
	At most 3 *	0.441	34.134	29.797	0.0149
	At most 4 *	0.373	17.849	15.495	0.0217
	At most 5 *	0.157	4.795	3.842	0.0285
Model 4	None *	0.921	217.260	95.754	0.0000
	At most 1 *	0.905	146.137	69.819	0.0000
	At most 2 *	0.696	80.126	47.856	0.0000
	At most 3 *	0.610	46.772	29.797	0.0002
	At most 4 *	0.385	20.397	15.495	0.0084
	At most 5 *	0.216	6.802	3.842	0.0091

### Residual test for models

As shown in Table 5, the 31 lags were selected for the models’ residual test and it was found that the AC and PAC values for models 1 and 2 were approaching 0 and non-significance using Q values, indicating non-autocorrelation for models 1 and 2 [35]. Further, the absolute AC and PAC values for models 3 and 4 are relatively larger and the Q values are significant, indicating autocorrelation for models 3 and 4; therefore, the least-squares method is appropriate for estimating models. To ensure comparability between the test results, the least-squares method was adopted to estimate the four models.

**Table 5:** The residual test results.

Observation periods(30)	Auto Correlation		Partial Correlation		Q-Stat		Prob.	
	Min	Max	Min	Max	Min	Max	Min	Max
Model 1	-0.283	0.357	-0.223	0.252	0.3357	21.001	0.240	0.859
Model 2	-0.168	0.278	-0.231	0.179	0.4814	19.705	0.488	0.902
Model 3	-0.323	0.777	-0.303	0.777	19.972	62.807	0.000	0.000
Model 4	-0.355	0.735	-0.376	0.735	17.908	56.092	0.000	0.002

### Model estimation

As shown in Table 6, only urbanization rate (UR) had a positive impact on suicide rate among urban men (UMSR) (a_5_ = 2.96, p = 0.0008), indicating that, for urban men, a higher urbanization rate caused a higher suicide rate; while marriage rate and per-capita GDP had negative impacts on the suicide rate (MR: a_1_ = -0.63, p = 0.0888; PGDP: a_4_ = -0.69, p = 0.0011). Therefore, the higher the marriage rate and the PGDP, the lower the suicide rate among urban men. Other two variables in model 1 had no significant impacts on suicide rate (a_2_ = -0.062,p = 0.949; a_3_ = 7.584,p = 0.390).For urban women, only urbanization rate (UR) had a positive impact on suicide rate (b_5_ = 4.56, p = 0.0001), indicating that the higher the urbanization rate, the higher the suicide rate; per-capita GDP had a negative impact on the suicide rate (b_4_ = -1.17, p = 0.0001), indicating that the higher the PGDP, the lower the suicide rate among urban women. Other three variables in model 2 had no significant impacts on suicidal rate (b_2_ = 0.745; p = 0.177; b_3_ = 0.656, p = 0.600; b_3_ = 0.635, p = 0.955).

**Table 6:** Linear regression estimation results for suicidal rate among urban men and urban women.

Model 1: Dependent variables(Suicidal rate among urban men (UMSR))					Model 2: Dependent variables(Suicidal rate among urban women (UWSR)				
	Coefficients	SD	T	P		Coefficients	SD	T	P
C01	-1.259	1.340	-0.940	0.357	C02	-2.648	1.738	-1.523	0.141
MR^(^^1^^)^	-0.628	0.354	-1.774	0.089	MR^①^	-0.745	0.459	-1.624	0.117
DR^(^^2^^)^	-0.062	0.952	-0.065	0.949	DR^②^	0.656	1.235	0.531	0.600
SR^(^^3^^)^	7.584	8.658	0.876	0.390	SR^③^	0.635	11.230	0.057	0.955
PGDP^(^^4^^)^	-0.700	0.188	-3.702	0.001	PGDP^④^	-1.172	0.244	-4.795	0.0001
UR^(^^5^^)^	2.964	0.776	3.817	0.001	UR^⑤^	4.564	1.007	4.532	0.0001
R^2^	0.466	Mean dependent var		1.782	R^2^	0.571	Mean dependent var		1.634
Adjusted R^2^	0.355	S.D. dependent var		0.311	Adjusted R^2^	0.481	S.D. dependent var		0.450
S.E. of regression	0.250	Akaike info criterion		0.241	S.E. of regression	0.324	Akaike info criterion		0.761
Sum squared resid	1.499	Schwarz criterion		0.522	Sum squared resid	2.521	Schwarz criterion		1.042
Log likelihood	2.379	Hannan-Quinn criter.		0.331	Log likelihood	-5.422	Hannan-Quinn criter.		0.851
F	4.187	Durbin-Watson stat		1.768	F	6.377	Durbin-Watson stat		1.748
Prob(F)	0.007				Prob(F)	0.001			

Note

(1) Marital rate.

(2) Divorce rate.

(3) Sex ratio.

(4) per capita GDP.

(5) Urbanization rate.

The adjusted R^2^ was approximately 35% for model 1; the R^2^ was 57% and the adjusted R^2^ is approximately 48% for model 2. The DW value for model 1 was 1.78 and approximately 2 for Model 2, indicating non-autocorrelation for the two models. The F values for the two models were significant, indicating good goodness-of-fit and explanatory power.

As shown in Table 7, the marital rate (c_1 =_ -0.50, p = 0.0187), p-capita GDP (c_4_ = -0.26, p = 0.014), and the rate of rural–urban labor migration in rural areas (c_5 =_ -0.20, p = 0.0891) had negative impacts on the suicide rate among rural men. That was, the higher the marital rate, p-capita GDP, and rate of rural–urban labor migration in rural areas, the lower the suicide rate among rural men. Other two variables in model 3 had no significant impacts on suicidal rate (c_2_ = 0.062, p = 0.882; c_3_ = 0.185). However, the marital rate (d_1_ = -0.37, p = 0.076), divorce rate (d_2_ = -091, p = 0.038), and rate of rural–urban labor migration in rural areas (d_5_ = -0.27, p = 0.003) were negatively associated with the suicide rate among rural women, namely, the higher the marital rate, divorce rate, and rate of rural–urban migration in rural areas, the lower the suicide rate among rural women. Other two variables in model 4 had no impacts on the suicidal rate (d_3_ = -5.367, p = 0.187; d_4_ = -0.067,p = 0.497).

**Table 7:** Linear regression estimation results for suicidal rate among rural men and rural women.

Model 3: Dependent variables(Suicidal rate among rural men (RMSR)					Model 4: Dependent variables(Suicidal rate among rural women (RWSR)				
	Coefficients	SD	T	P		Coefficients	SD	T	P
C_03_	6.611	0.475	13.928	0.0000	C_04_	4.766	0.472	10.090	0.0000
MR(^1^^)^	-0.503	0.199	-2.523	0.019	MR^①^	-0.369	0.198	-1.858	0.076
DR^(^^2^^)^	-0.062	0.416	-0.150	0.882	DR^②^	-0.912	0.414	-2.201	0.038
SR(3)	-5.429	3.973	-1.366	0.185	SR^③^	-5.367	3.954	-1.357	0.187
PGDP^(^^4^^)^	-0.258	0.097	-2.649	0.014	PGDP^④^	-0.067	0.097	-0.690	0.497
RUMR^(^^5^^)^	-0.207	0.117	-1.772	0.089	RUMR^⑤^	-0.267	0.116	-2.302	0.030
R^2^	0.960	Mean dependent var		2.639	R^2^	0.919	Mean dependent var		2.636
Adjusted R^2^	0.952	S.D. dependent var		0.522	Adjusted R^2^	0.902	S.D. dependent var		0.363
S.E. of regression	0.114	Akaike info criterion		-1.321	S.E. of regression	0.114	Akaike info criterion		-1.331
Sum squared resid	0.314	Schwarz criterion		-1.041	Sum squared resid	0.311	Schwarz criterion		-1.051
Log likelihood	25.816	Hannan-Quinn criter.		-1.231	Log likelihood	25.962	Hannan-Quinn criter.		-1.241
F	115.876	Durbin-Watson stat		0.998	F	54.159	Durbin-Watson stat		1.341
Prob(F)	0.0000				Prob(F)	0.0000			

Note

Marital rate.

Divorce rate.

Sex ratio.

per capita GDP.

Rate of rural–urban labor migration to the total labor population in rural areas.

The R^2^ and adjusted R^2^ for Model 3 was approximately 96%, and 95%, respectively, and the R^2^ and adjusted R^2^ for Model 4 were approximately 92% and 90%, respectively; the higher R^2^ might indicated a multicollinearity problem. The DW value for Model 3 was 0.99 and that for model 2 is 1.34, indicating a certain autocorrelation problem, which was in accordance with the results from the residual test.

## Discussion

### What does marital event mean to suicidal rate in gender context?

The results indicated marriage hold different meanings to people of different genders that live in different areas. Marriage rate was a significant protective factor for urban men, rural men, rural women except for rural women. This finding was basically in accordance with Durkheim’s viewpoints and other similar studies, namely that marriage might reduce suicidal risks, especially for men; single men are more likely to face suicidal risks [11,15–21].

The results indicated that divorce also hold different meanings to people of different genders that live in different areas. Divorce rate was a significant protective factor only for rural women, but it was non-significant for urban men, urban women, and rural men. This finding was basically in accordance with Durkheim’s viewpoints and other similar studies [11,15,16] but conflicted with some studies on the relationship between divorce and suicide, namely that divorce and widowhood would apparently reduce the psychological welfare of men and women and induce negative psychological problems such as stress and depression [17–21,36].

### What do marital events mean to suicidal rate in rural-urban context?

Most interestingly, it was noteworthy in this study that marriage and divorce played same roles among Chinese urban and rural men but different roles among Chinese urban and rural women, especially that marriage rate and divorce rate had significant impacts on suicide rate among rural women but no impacts on suicide rate among urban women. The possible reason was that urban women were more and more independent economically and psychologically, therefore, marriage and divorce might play less and less important roles in their lives [37]; while for rural women, marriage and divorce still occupied a big space in their lives. A happy marriage would help rural women reduce their psychological pressure; while a high divorce rate was coupled with an increase in socioeconomic development and women’s status [38]. Divorce also helped rural women to end unhappy marital relationships and improve the level of their psychological health; therefore, both of them were helpful to reduce the suicide rate among rural women [12–14].

### What does marriage squeeze mean to suicidal rate in rural-urban context?

The results indicated that the sex ratio had no any significant impact on suicidal rate among Chinese people of different genders in different regions. A possible reason was that sex imbalance and marriage squeeze constitute the variables at the macro level. It was difficult for individuals at the micro level to have direct feelings regarding marriage squeeze; however, they may have direct feelings regarding marriage and divorce, which may influence their psychological feelings and behaviors. Other studies on sex imbalance and marriage squeeze indicated that sex imbalance and marriage squeeze were commonly measured by individual variables such as age, marital status, and difficulties in getting married [39]. Existing studies also stated that age, marital status, and difficulties in getting married have apparent impacts on the quality of life at the individual level [39]. But in this study, the direct evidence on the relationship between the marriage squeeze and suicidal behaviors among Chinese was obtained.

### What does economic development mean to suicidal rate in rural-urban context?

For men and women in urban areas, p-capita GDP was a protective factor against suicide rate, which was possibly because p-capita GDP was directly associated with individual economic status, and improving economic statuses helps men and women in urban areas to relieve and resist pressures, which decreased the suicide rate [40]. For men rather than women in rural areas, the p-capita GDP was also a protective factor against suicide rate. A possible reason was that an increase in p-capita GDP may improve the individual economic status of men who lived in rural areas and made a greater contribution to the family income and enjoyed the higher economic power and benefits than women, which would lead to a decrease in the suicide rate among rural men [40].

### What does the social development mean to suicidal rate in rural-urban context?

The urbanization rate and the rate of rural–urban labor migration in rural areas were adopted to represent the socioeconomic status in urban and rural areas, respectively; The urbanization rate was a risky factor to the suicide rate among urban men and women, which conflicted with the existing findings that urbanization helps reduce the suicide rate [41]. A possible reason was that the increase in urbanization rate mean that more rural population had left their hometown to live in the urban areas, which might increase the suicide rate in urban areas, while urbanization would cause cultural conflicts between urban and rural areas, which increased the suicide rate [42]. The rate of rural–urban labor migration to the total rural population was a protective factor for the suicide rate among rural men and women, which was in accordance with the existing research [5]. The possible reason was that rural–urban migration results in new lifestyles and concepts, such as the improvement in women’s economic status and marital autonomy, which would reduce the suicide rate among rural men and women [43].

## Conclusion and prospects

From the results and discussion above, we drew some conclusions as follows:

### Conclusion 1: Both marriage and divorce rates were protective factors for the suicide rate in rural and urban Chinese, but had different meanings in urban and rural areas

Marriage rate was a protective factor for suicide rate among urban men, rural men, and rural women, but it had no impact among urban women. The divorce rate was a protective factor for suicide rate among rural women but had no impact on suicide rate among urban men, urban women, and rural men.

### Conclusion 2: Economic development was a protective factor for urban and rural Chinese, except for women in rural areas

Per capita GDP was a protective factor against suicide rates among urban men, urban women, and rural men, but it had no impact on the suicide rate among rural women.

### Conclusion 3: Social development had different impacts on the suicide rate among rural and urban Chinese

The urbanization rate was a risky factor for suicide rates among urban men and women; however, the rate of rural–urban labor migration in rural areas was a protective factor against the suicide rate among rural men and women.

### Limitations

This study focused on the suicidal rate and its associated factors in Chinese background, and partly supported the classic theory proposed by Durkheim (2010) and proved in other studies [11,15,16], validating the protective function of marriage and divorce for different people. However, it was still uncertain in other social and cultural background [17–21,36]. Even for this study, there were some limitations should be considered and improved in the future as followed:

### Data limitations

The data used in this study, such as suicidal, divorce, and marriage data, only covered the national level; therefore, the data at the provincial and area levels were lacking, which did not reflect the differences between provinces and areas. Further, the survey data at the micro level were lacking, which made it difficult to study the impacts of the multi-dimensional attributes of marriage on suicidal behaviors among various groups in urban and rural areas. A future study should be conduct a survey covering men and women residents in urban and rural areas with the multi-dimensional attributes of marriage as the explanatory variable, to collect data on family, marriage, and suicidal intentions and thus further analyze the relationship between micro-level marriage and individual behaviors.

### Methodological limitations

Owing to the limitations of macro-level data size, only a relatively simple linear regression methodology was adopted to estimate the parameters; therefore, some important variables could not be included in the models. Meanwhile, the marital status at the micro level might affected suicidal behaviors through quality of marriage as the mediator, which was hardly reflected in the macro-level study. As an ecology problem, only the sociological methods rather than ecology methods were adopted in this paper due to the limitations in data, which may also bring bias results and conclusions. Future studies should construct a multi-level mechanism model at the micro-level to investigate suicidal behaviors among urban and rural residents by gender and collect data for testing the model.

### Limitations in discussions

This study analyzed the associations between several rates and suicide rates, including marital events (marriage rate, divorce rate and marriage squeeze), economic development, and social development. It was hard to explain all these rates in one article, and it made that some explanations were hard in deep way in content of discussions.

## Supporting information

S1 File (SAV)
